# Impact of using infographics as a novel Just-in-Time-Teaching (JiTT) tool to develop Residents as Teachers

**DOI:** 10.15694/mep.2020.000289.2

**Published:** 2021-09-22

**Authors:** David Orner, Alice Fornari, Sarah Marks, Timothy Kreider

**Affiliations:** 1 Department of Radiation Medicine; 2 Department of Radiation Medicine; 3 Department of Science Education; 4 Department of Science Education; 5 Department of Psychiatry; 6 Department of Psychiatry

**Keywords:** Just in Time Teaching, Resident as Teachers, Infographic

## Abstract

This article was migrated. The article was marked as recommended.

**Introduction:** Resident as Teacher (RaT) curriculum continues to be recognized as a critical component of residents’ education. However, in busy clinical workplaces, there are time constraints limiting formal training for RaT. This study aimed to assess the engagement and acceptability of Just-in-Time-Teaching (JiTT) infographics, a novel RaT tool that provides digestible evidence-based knowledge & skills at the time & place where teaching happens.

**Methods:** The study focused on residents and faculty between July – September 2019, across 12 residency programs in six specialties. JiTT infographics were distributed weekly for six weeks. Engagement of residents was measured by open rates of infographics. Acceptability was evaluated using mixed-methods analysis of a questionnaire at the conclusion of the study, and a focus group with appointed resident and faculty champions. Descriptive statistical analyses were applied to ascertain preliminary results.

**Results:** Overall, 76.2% (198/260) of residents opened infographics, with an average engagement rate of 64.9% Analysis of acceptability evaluations revealed infographics to be effective and had a positive impact on teaching style.

**Discussion and Conclusion:** JiTT infographics function as an educational approach to support teaching strategies in the busy clinical setting. It can be adapted across diverse programs and integrated into a teaching toolbox for residents and faculty.

## Introduction

Resident physicians play an instrumental role to teach clinical skills to junior residents and medical students ( Bree *et al.*, 2014; Hill *et al.*, 2012). Several studies have found that residents spend at least 25% of their time teaching, and one-third of medical students’ clinical skills and knowledge is attributable to their guidance ( Bing-You and Sproul, 1992; Seely *et al.*, 1999; Montacute *et al.*, 2016). To improve resident teaching skills, Canadian and United States accreditation bodies require that residents be formally trained to teach students ( ACGME, 2020; Frank, Snell and Sherbino, 2015). Increasingly, specialties have adapted Resident as Teacher (RaT) initiatives into their residency programs ( Al Alchkar *et al.*, 2017); ( McKeon *et al.*, 2019; Alaska *et al.*, 2019; Ravichandran *et al.*, 2019) by incorporating a variety of curricula and teaching methodologies ( Habermehl, Habermehl and Kim, 2018; Tan *et al.*, 2017; Watkins *et al.*, 2017; Arya *et al.*, 2018; Ricciotti *et al.*, 2017). However, due to increased clinical responsibilities and time constraints, it is a challenge for programs to allocate enough time to enhance their skills ( Ravichandran *et al.*, 2019; Habermehl, Habermehl and Kim, 2018; Lacasse and Ratnapalan, 2009).

Recently, information graphics, or infographics, have become an increasingly popular format for communication in both teaching and learning due to their ability to capture attention, engage and make complex concepts and/or knowledge easily and quickly understandable ( Ozdamli and Ozdal, 2018; Ray Chaudhury, 2019). Well-designed infographics can be understood faster than text alone and are twice as likely to be retained ( Scott *et al.*, 2016). Many studies ( Clark and Mayer, 2016; Green and Myers, 2010; Carney and Levin, 2002) reported the incorporation of visuals can enhance the learning of educational information while reducing cognitive load ( Mayer and Moreno, 2003; Martin, 2014). Healthcare professionals have begun to embrace images and graphics along with mobile technology (Thoma *et al.*, 2018) to communicate. To our knowledge, there is a dearth in published data (Thoma *et al.*, 2018; Chin *et al.*, 2019; Dowhos *et al.*, 2021) on using infographics to train residents on how to teach.

With a growing demand to enhance the existing curriculum among our residents and faculty, concomitant with requirements set forth by the accreditation bodies ( ACGME, 2020; Frank, Snell and Sherbino, 2015) we investigated the engagement and acceptability of Just-in-Time-Teaching (JiTT) infographics, a novel approach adaptable to diverse RaT programs that deliver evidence-based clinically relevant teaching tips. The Kirkpatrick’s ( Frye and Hemmer, 2012) model of evaluation was considered to assess the engagement and acceptability of this approach. We hypothesized that JiTT infographics would promote a culture of effective teaching via self-directed learning and provide a faculty development framework for residents and faculty across specialties.

## Methods

This study took place during six-week rotations from July to September 2019 across 12 residency programs in six clinical specialties (Internal Medicine (IM), Neurology, Obstetrics/Gynecology (OB/GYN), Surgery, Psychiatry, and Pediatrics). There were 260 residents and 237 faculty from five tertiary-care hospitals within a large suburban health system. Faculty inclusion was based on feedback received from a prior pilot study among residents with a goal for faculty to help reinforce engagement with JiTT content among residents.

### Description of JiTT Infographics

JiTT infographics [https://libguides.hofstra.edu/mededresources/teachingresources] were implemented as a pedagogical approach to provide teaching tips when residents need them to engage with students. Infographics included both foundational clinical teaching principles (e.g., bedside teaching, learning huddle, and 5-microskills) (Supplementary File 1) and specialty-specific (e.g., how to conduct an abdominal exam, evaluate for rupture of membranes/amniotic fluid, and review neurologic imaging) (Supplementary File 2). Content for the foundational clinical teaching principles were developed by the principal investigator (AF). Specialty specific content was developed by our residents and faculty champions.

Prior to dissemination, program directors of participating residencies were recruited and asked to appoint a core faculty and resident champion to support the new initiative. The responsibilities of each champion were to provide insight regarding the logistics of delivery, identify modality for distribution based on the programs’ communication culture, and prepare specialty-specific teaching content. With the assistance of a program manager and a faculty developer project manager part of our centralized faculty development administration infographics were created using Canva Pro (Canva, Ltd, Australia), a premium graphic design platform.

### Outcomes measured

Resident engagement was quantified through OpenMoves (an email marketing platform) by analyzing the rate of opened emails /total residents in each program. Subsequently, the acceptability of the study was analyzed using mixed-method analysis consisting of (1) a semi-structured focus group conducted with 36 resident and faculty champions to discuss end-user feedback, and (2) electronic resident and faculty surveys (Supplemental File 3), which were adapted from a previously published survey ( Watkins *et al.*, 2017); to collect data to support program evaluation at Kirkpatrick level 1 (satisfaction) and 2 (learning).

### Statistical Analysis

To ensure adequate data was collected we clustered data by clinical specialty. Quantitative data were analyzed using Prism 8 (GraphPad Software San Diego, CA). Differences between weekly engagement rates were analyzed using Chi-Square with Yates correction. p < 0.05 was considered statistically significant.

Descriptive analyses (count, mean, standard deviation (SD), and percentage as appropriate) were used to assess acceptability metrics and outcomes. Qualitative survey questions and narrative text from the focus group were reviewed and coded independently using Microsoft Excel by two members of the research team (KS and DO) using conventional content analysis ( Hsieh and Shannon, 2005). This research study was deemed exempt by the Northwell Health Institutional Review Board (IRB) as a supplementary educational tool.

## Results

### Engagement Results

Over the 6-week period, 198 out of 260 (76.2%) residents opened weekly emails with an average engagement rate of 65.7%. The average specialty engagement rates were highest among pediatrics (71.4%) compared with neurology (67.8%), psychiatry (64.5%), IM (62.7%), surgery (62.2%) and OB/GYN (60.8%).

Further analysis showed that while there was an 8.5% decline in weekly average engagement rate for all residency programs from 68.5% (178/260) in week 1 to 60.0% (156/260) in week 6; it was not statistically different (p = 0.054).

### Acceptability Results

A total of 44 (22.2%) residents and 60 (25.3%) faculty that opened JiTT completed the survey. Table 1 shows the results about the acceptability of JiTT infographics for residents.

**Table 1:** Self-Reported Resident Survey Results (N= 44)

	Residents who responded "highly effective" or "moderately effective" ^a^ No. (%)	Mean [SD]
Rate the JiTT infographics tip sheets day of delivery	29 (65.9)	3.23 [0.94]
Rate the JiTT infographics tip sheets time of delivery	29 (65.9)	3.20 [0.95]
How effective were the (general) JiTT infographic tip sheets in...		
Teaching with Limited Time	30 (68.2)	3.09 [0.80]
Setting Goals & Expectations	30 (68.2)	3.08 [0.86]
Directed Observation Through Teaching	27 (61.4)	3.10 [0.82]
Using Questioning as an Effective Tool for Teaching	31 (70.5)	3.05 [0.88]
Bedside Teaching	29 (65.9)	3.08 [0.94]
SFED Model of Feedback	20 (45.5)	3.28 [0.88]
How effective were your JiTT infographic tip sheets specific to your clinical specialty?	32 (72.7)	3.05 [0.85]

*Abbreviations: JiTT, Just in Time Teaching; SFED, Self-Assessment Feedback/Facts Encouragement Direction*

^a^ *Residents rated each of these items using a 4-point Likert scale (1 = not effective, 2 = slightly effective, 3 = moderately effective, 4 = highly effective)*

As shown, 20 (45.5%) responded the role of resident as teach teacher “is one of my primary responsibilities”, 42 (90.9%) felt that opening the infographics prompted them “to think about teaching”, 33 (75.0%) found the frequency to be just right, and 31 (70.4%) changed their teaching styles as a result.

Furthermore, 50 (83.3%) faculty felt the JiTT infographics were a useful resource to enhance faculty teaching skills in the clinical environment, whether with students or residents. 29 (48.3%) encouraged residents to review the JiTT infographics.

Despite these promising results, these findings only represented the acceptability and satisfaction among the limited end-users. However, further thematic analysis of qualitative data confirmed these findings ( Table 2) with faculty solidifying the value of JiTT as well as their involvement to engage residents in the program and most important role model and develop resident skills to utilize the infographic content.

**Table 2:** Themes about residents' learning experience, with JiTT infographics

Theme	Subtheme	Representative quote
Overall perception of JiTT:		
Positive	Informative	*It gave me ideas of what to go over with the students if students were present on my rotation.*
Concise	*The succinctly displayed information that could be utilized to enhance the teaching/learning experience.*
Good Reminders	*Served as a reminder to always think about teaching*
Negative	Too broad/general	*Could do with more specific examples, examples of good vs bad strategies*
Became repetitive	*Grew repetitive and eventually did not look at them weekly*
Improvements	More integrative	*Could be built into the curriculum in a more interactive way, weekly emails tend to get ignored*
Content	*I like the overall concept. I think more specific and advance topics could be sent out. Going over the abdominal exam or how to ask questions to the students is not beneficial. I want to teach them information that they can use for their examinations or future job. I want to let them take the lead on minor procedures (e.g., NG tubes, incision and drainage, etc.) and teach them specifics (how to diagnose and treat surgical conditions [cholecystitis, peripheral vascular disease], which patients need operations, etc.).*

*Abbreviations: NG, nasogastric*

### Discussion

In the current US and Canadian graduate medical education curriculums, residents have inadequate opportunity to learn how to teach due to limited time away from patient care responsibilities ( Ravichandran *et al.*, 2019; Arya *et al.*, 2018; Sward, Ellis and Mercado, 2020). Kirkpatrick’s Evaluation Model guides programmatic evaluation of the impact of JiTT infographics on resident’s perception of their role as clinical teachers and faculty as role models. JiTT infographics has allowed us to positively influence resident’s perception of their teaching skills and reach Kirkpatrick level 1(satisfaction) and level 2 (learning).

This study confirmed that infographics are adaptable to an array of clinical specialties and can be successfully implemented as a technology-enhanced pedagogical tool (Thoma *et al.*, 2018; Chin *et al.*, 2019; Dowhos *et al.*, 2021) providing residents with the capacity to apply teaching skills ‘just in time’ in the clinical setting. A positive outcome was faculty satisfaction with the JiTT program and their ability to role model teaching skills, which leads to a positive aura of the JiTT program in the clinical environment. This also aligned with a recommendation from residents in an earlier pilot program that faculty must receive the same content as residents.

Finally, our system to launch the JiTT program included clinical specialty specific resident and faculty champions to assure the messaging about JiTT came from peers. This proved to be a very valuable starting point for this program to launch. From the focus group and surveys, we were able to ascertain several ways the JiTT infographics can improve its acceptability and usefulness which include: mini-podcast links attached to infographics making content audible and providing examples for each teaching tip; visual reminders of infographics in strategic locations, where they could be seen by faculty and residents; and changes in dissemination frequency by shortening the number of distribution weeks, use of a consortium-based approach (e.g. a blend of general and specialty-specific JiTT infographics). Recently, all infographics became available via a software application (App) (Supplementary File 4) to download on both iOS and Android devices. The adaptability of all content created for email distribution to electronic devices increases international end-user accessibility, especially since the JiTT infographic App is open access.

## Limitations

There are several limitations to our study. First, selection bias may influence the survey data -- users who opened the JiTT infographics may be more likely to complete an emailed survey. Second, our study focused on the short-term impact on residents and faculty, specifically receptiveness to a new form of clinical education using visuals and technology. Our current evaluation was unable to measure the longitudinal impact on resident teaching or medical student learning. To determine an overall causative relationship between an educational innovation and teaching and learning outcomes may be difficult due to an array of confounding variables in our diverse clinical environments and the uniqueness of each clinical program. In addition, we need to further use our resident and faculty champions to understand program culture that impacts infographic initial and consistent open rates. These initial findings are a valuable first step and further studies will refine logistics and measurement of longer-term impact of infographics to determine RaT and faculty best practices in busy clinical environments.

## Conclusion

In a mixed-method experiential study, we provided preliminary evidence that the creation of JiTT infographics is a viable technologically-enhanced strategy to develop and promote RaT in the clinical setting. Analysis showed the infographics were effective for 2 levels of Kirkpatrick to evaluate educational interventions. This adaptation to technology-assisted education that considers time sensitive delivery is especially important in light of the digital adeptness of residents and restricted duty hours. Most important we can say with certainty that the JiTT infographic program can be incorporated into diverse busy teaching and clinical settings. In addition it is accessible on open-access technological devices available in the clinical setting to ensure “just in time” access and application.

## Take Home Messages

• JiTT infographics provides trainees and faculty with visually accessible evidence-based teaching tips to enhance skills ‘just in time’ in the clinical setting.
• Preliminary results illustrate this approach can be an effective way to engage trainees and faculty to teach medical students and junior trainees.
• The ‘just in time teaching tip’ is adaptable across clinical specialties and can be foundational and clinically specific.

## Notes On Contributors

David Orner MPH, is a research coordinator in the Department of Radiation at Donald and Barbara Zucker School of Medicine at Hofstra/Northwell, New York. ORCID ID: https://orcid.org/0000-0002-2208-196X

Dr. Alice Fornari EdD; RDN, is the Vice President of Faculty Development at Donald and Barbara Zucker School of Medicine at Hofstra/Northwell, New York. ORCID ID: https://orcid.org/0000-0001-5475-2732

Dr. Sarah M. Marks MD., is a Psychiatry Resident (PGY3) at Donald and Barbara Zucker School of Medicine at Hofstra/Northwell, New York. ORCID ID: https://orcid.org/0000-0002-9291-789X

Dr. Timothy R. Kreider MD., PhD, is the Clerkship Director of Psychiatry at Donald and Barbara Zucker School of Medicine at Hofstra/Northwell, New York. ORCID ID: https://orcid.org/0000-0002-0934-8244
