# Supplementary material for: Impact of using infographics as a novel Just-in-Time-Teaching (JiTT) tool to develop Residents as Teachers
Source: MedEdPublish (2016). 2021 Sep 22;9:289. Originally published 2020 Dec 23. [Version 2] doi: 10.15694/mep.2020.000289.2 (PMC10697492; doi:10.15694/mep.2020.000289.2)
Supplement: Peer review history

# Reviewer report: Reviewer response for version 2

**DOI:** 10.21956/mep.20288.r31504

**Published:** 2021-10-13

**Authors:** Haipeng Xiao

**Affiliations:** 1 The First Affiliated Hospital, Sun Yat-sen University

This review has been migrated. The reviewer awarded 4 stars out of 5 Thank you for addressing the issues I have raised for the previous version. I have enjoyed reading the updated manuscript. My sincere wishes for the future works of these authors.

Reviewer Expertise:

NA

No decision status is available

# Reviewer report: Reviewer response for version 2

**DOI:** 10.21956/mep.20288.r31503

**Published:** 2021-10-09

**Authors:** Anita Samuel

This review has been migrated. The reviewer awarded 4 stars out of 5 This is an interesting approach to disseminating information in time-constrained environments. Thank you for sharing the JiTT resources that were created. They provide a clear idea of how you have used infographics.It would be helpful to include some information about how these resources were deployed. There’s a passing reference in the Results to “weekly emails” which is the only information provided about deployment. One limitation that should be noted is that the evaluation at Kirkpatrick level 2 is based on self-reported data. There is no objective assessment of learning.

Reviewer Expertise:

NA

No decision status is available

# Reviewer report: Reviewer response for version 2

**DOI:** 10.21956/mep.20288.r31505

**Published:** 2021-09-24

**Authors:** Ken Masters

**Affiliations:** 1 Sultan Qaboos University

This review has been migrated. The reviewer awarded 4 stars out of 5 The authors have addressed the concerns I raised about Version 1 of their paper, and it is now an improved reading experience. I look forward to future research that they may perform in this area.

Reviewer Expertise:

NA

No decision status is available

# Reviewer report: Reviewer response for version 1

**DOI:** 10.21956/mep.19145.r27681

**Published:** 2021-02-28

**Authors:** Haipeng Xiao

**Affiliations:** 1 The First Affiliated Hospital, Sun Yat-sen University

This review has been migrated. The reviewer awarded 5 stars out of 5 Few would dispute that teaching ability is an important requirement for residents. This is why Resident as Teacher (Rat) curriculum is considered a significant part of the resident’s training. This paper shows us an important and interesting approach to facilitate the development of RaT, using infographics as a novel Just-in-Time-Teaching (JiTT) tool, which helps the residents to teach ‘just in time’ in the clinical encounter. The study is instructive and practical, using a mix-method analysis, with adequate information clearly describing the JiTT infographics. Thank you very much for sharing the study of this novel approach to RaT curriculum. It is very helpful and inspiring. Although introduction in more detail to both the origin and development of Just-in-Time-Teaching may be needed to facilitate readers’ understanding, this well-written paper should be recommended to among residents, faculty, and administrators of medical education to arouse their attention to the development of RaT as well as consideration about how to improve RaT education in the residency training program in China with an acceptable RaT curriculum.

Reviewer Expertise:

NA

No decision status is available

# Reviewer report: Reviewer response for version 1

**DOI:** 10.21956/mep.19145.r27679

**Published:** 2021-02-24

**Authors:** Ken Masters

**Affiliations:** 1 Sultan Qaboos University

This review has been migrated. The reviewer awarded 4 stars out of 5 An interesting paper on using infographics as a just-in-time-teaching tool to develop residents as teachers. The paper clearly outlines the problem to be solved, as the need to train Residents as Teachers, but the lack of time because of increased clinical duties. The proposed solution was to distribute infographics, and to measure the engagement and perceived value. The examples supplied (both in the supplementary files and the link) give a good insight into the type and scope of work covered by the infographics.Overall, and mindful of the limitations, the paper is well-written, and describes a nice solid piece of research on a useful topic.Some areas that could benefit from attention:• Only the name of the software used is supplied. As there are different versions (with different costs and potentials), more details should be given.• The work and time and human resources should be described. If the paper is to have value to those who would like to follow this example, this information is crucial.• Which software, if any, was used to theme the qualitative data?• The Discussion is given as a single block of text, and it would be more easily readable if this were broken into smaller paragraphs.Thank you for a good paper. I look forward to version 2 that is strengthened by addressing these items and those raised by the other reviewers.

Reviewer Expertise:

NA

No decision status is available

# Reviewer report: Reviewer response for version 1

**DOI:** 10.21956/mep.19145.r27682

**Published:** 2021-01-10

**Authors:** Alexandra Webb

**Affiliations:** 1 Australian National University

This review has been migrated. The reviewer awarded 4 stars out of 5 Thank you for sharing your initiative to promote the development of education skills of residents. It was interesting to learn about the practicalities of developing and implementing the infographics and the responses of users. Please see a few suggestions for the paper below.1. It would be helpful to the reader to clarify the specific issue – is there insufficient time for formal instruction or teaching experience/practice or something else? – to define the gap that the infographics were designed to fill.……… it is a challenge for programs to allocate enough time to enhance their skills (Ravichandran et al., 2019; Habermehl, Habermehl and Kim, 2018; Lacasse and Ratnapalan, 2009). 2. Provide some more evidence and supporting citations here e.g. Mayers’ Principles of Multimedia LearningWell-designed infographics can be understood faster than text alone and are twice as likely to be retained (Scott et al., 2016).3. Clarify details about the infographic creation e.g. Who developed the infographics –the residents or faculty or both? Who determined the format, content, design etc.? Who provided an overview/review to check content, ensure consistency, quality control?4. Consider rewording the following:a. Difficult sentence to read: Despite these promising results, these findings were only represented the acceptability and end-user satisfactionb. Clarify if this refers to a software application (app): and finally the future use of an APP to store all infographics5. Incorporate a brief description of how and when residents get opportunities to receive feedback on their teaching from peers and/or faculty

Reviewer Expertise:

NA

No decision status is available

# Reviewer report: Reviewer response for version 1

**DOI:** 10.21956/mep.19145.r27680

**Published:** 2020-12-24

**Authors:** Elisabeth Schlegel

**Affiliations:** 1 Donald and Barbara Zucker School of Medicine at Hofstra/Northwell

This review has been migrated. The reviewer awarded 4 stars out of 5 The adage "A picture is worth a thousand words" holds also true in medical education. Distilling multiple and complex ideas into an image is an art allowing the viewer – here a teaching resident - to quickly comprehend and internalize an educational concept. The authors present the research on the effectiveness of their masterfully crafted infographics termed “JiTTs”, teaching tip sheets, which have been prepared for busy residents to convey a just-in-time -teaching point.This paper provides valuable introspection into using infographics as RaT components, including development, recruitment of champions for buy-in, dissemination, and generation of feedback data. I enjoyed reading the paper and appreciate the infographics provided with the data. One idea that comes to mind, is, using the easy-to-comprehend tip sheets as prompts for rounds and other educational meetings, and generate discussion on the topic. Overall, this is a great approach to inform education under time constraints which are transferable into any field of education with the ability to use visuals.

Reviewer Expertise:

NA

No decision status is available
